# Brevetoxin Dynamics and Bioavailability from Floc Following PAC-Modified Clay Treatment of *Karenia brevis* Blooms

**DOI:** 10.3390/toxins17110560

**Published:** 2025-11-13

**Authors:** Nicholas R. Ohnikian, Christopher D. Sibley, R. Ben Freiberger, Kristen N. Buck, Alyssa Myers, Samantha Harlow, Donald M. Anderson, Richard Pierce, Jennifer H. Toyoda

**Affiliations:** 1 Ecotoxicology Research Program, Mote Marine Laboratory, Sarasota, FL 34240, USA; nohnikian@mote.org (N.R.O.); csibley@mote.org (C.D.S.); alyssa.meyers@spartans.ut.edu (A.M.); sharlow@mote.org (S.H.); rich@mote.org (R.P.); 2 College of Earth, Ocean, and Atmospheric Sciences, Oregon State University, Corvallis, OR 97331, USA; freiberr@oregonstate.edu (R.B.F.); kristen.buck@oregonstate.edu (K.N.B.); 3 Woods Hole Oceanographic Institution, Woods Hole, MA 02543, USA; danderson@whoi.edu

**Keywords:** *Karenia brevis*, brevetoxin, harmful algal blooms, HABs, flocculation, floc, red tide, modified clay

## Abstract

Harmful algal blooms (HABs) caused by the dinoflagellate *Karenia brevis* present serious ecological and public health concerns due to the production of brevetoxins (BTX). Clay flocculation and sedimentation of cells, particularly with polyaluminum chloride (PAC)-modified clays, is a promising HAB mitigation approach. This study evaluated the efficacy of Modified Clay-II (MCII), a PAC-modified kaolinite clay, in reducing *K. brevis* cell abundance in mesocosm experiments and examined the bioavailability of BTX potentially released from settled floc back into the water column and sediment over the first 72 h after treatment. Additionally, we quantified trace metals in benthic clams (*Mercenaria mercenaria*) exposed to the floc post-treatment to assess metal accumulation and potential toxicological effects from MCII application. MCII treatment (0.2 g/L) resulted in a 91% reduction in *K. brevis* cell density and a 50% decrease in waterborne brevetoxins after 5 h. Brevetoxins accumulated in sediment post-flocculation, with BTX-B5 emerging as the dominant congener. Clams exposed to MCII-treated floc showed comparable tissue BTX levels to controls and significantly elevated aluminum concentrations, though without mortality. The aluminum accumulations in this study do not raise concerns for the health of the clams or the humans who eat them, given other dietary exposures. These findings support the potential of MCII for HAB mitigation while underscoring the need for further evaluation of exposure risks to all benthic species.

## 1. Introduction

**Figure 1:**
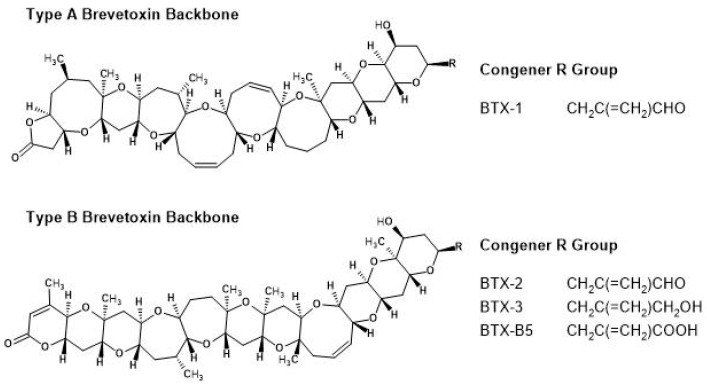
Chemical structures of Type A brevetoxin backbone congener BTX-1 and Type B brevetoxin backbone congeners BTX-2, BTX-3, and BTX-B5.

The development of harmful algal blooms (HABs) occurs when algal populations grow rapidly and uncontrollably, leading to significant environmental disruption and potential mass die-offs of entire ecosystems [1,2]. Beyond these environmental impacts, certain HABs release toxic compounds into the water, posing serious health risks or even causing death for wildlife, livestock, and humans who are exposed to toxins by water contact, inhalation, or diet [3,4,5,6]. *Karenia brevis* (*K. brevis*, G. Hansen and Moestrup (formerly *G. breve*, C.C. Davis)) is one of the most well-known and widespread toxin-producing marine HAB species. This microscopic, single-celled dinoflagellate is commonly found in the Gulf of Mexico and is best recognized as the organism responsible for the “Florida Red Tide,” a recurring HAB event that also occurs in other parts of the world. Red Tide blooms can persist for weeks to years and are associated with extensive marine life die-offs, resulting in millions of dollars in economic losses for coastal communities globally [7]. As shown in Figure 1, *K. brevis* naturally produces a group of potent cyclic polyether neurotoxins collectively referred to as brevetoxins (BTX) [8,9]. Once inhaled or ingested, these neurotoxins bind to voltage-gated sodium ion channels, disrupting normal nerve signaling and leading to a range of adverse effects including respiratory, neurological, and gastrointestinal symptoms in humans and other organisms [10,11,12,13,14,15,16]. Although extensive research into mitigation strategies aimed at reducing *K. brevis* cell impacts has been conducted in recent years, direct bloom control strategies remain among the least advanced. This highlights the ongoing need for continued development and evaluation of new mitigation approaches and their toxicological effects.

Current mitigation strategies for *K. brevis* can be broadly categorized into three approaches: chemical [17,18,19], biological [20,21], and physical [22,23,24]. Chemical methods involve the use of synthetic or naturally derived agents designed to disrupt cellular integrity or metabolic function, ultimately leading to cell death. While some chemical treatments have shown promise in laboratory settings, their large-scale application raises public and environmental concerns, particularly regarding potential long-term ecological impacts on marine and freshwater systems. Biological mitigation employs living organisms to control *K. brevis* populations in a more environmentally sustainable manner. This includes microorganisms that either consume algal cells as a food source or exhibit natural algicidal properties. However, the broader adoption of biological approaches has been limited by challenges in scaling up organism production for widespread use, as well as practical issues related to storage, transport, and delivery to affected sites. In contrast, physical mitigation strategies such as water filtration, surface skimming, and induced flocculation to manually remove *K. brevis* cells from the water have shown great promise. These methods have gained global attention due to their demonstrated effectiveness, scalability, lower cost, and reduced environmental impact compared to chemical and biological alternatives [25].

Clay flocculation has been explored as a mitigation strategy for HABs since the 1980s, particularly in East Asia, where recurring blooms have had significant ecological and economic impacts surrounding aquaculture [26,27,28]. This method involves adding fine clay particles to bloom-affected waters, which bind to algal cells and form aggregates (flocs) that settle out of the water column where conditions are unsuitable for further algal growth. The physical removal of algae through sedimentation helps reduce bloom intensity and associated toxin production [29]. This approach has been applied for decades in countries such as China and South Korea, however its implementation in the United States remains under active investigation [22,23,25,30,31].

Over time, different formulations of natural and modified clays, such as kaolinite and loess, have been tested to optimize flocculation efficiency and minimize environmental side effects [31]. One of the most promising modified clays currently under investigation for *K. brevis* mitigation is polyaluminum chloride (PAC)-modified kaolinite clay [22,29]. This approach involves mixing kaolinite clay with polyaluminum chloride, an inorganic polymer commonly used in drinking water treatment [25]. PAC enhances algal flocculation primarily by altering the surface charge of clay particles to be more positive, making them highly attractive to negatively charged *K. brevis* cells, thus promoting floc formation and aggregation. Its polymeric aluminum species also form cationic patches that bridge between clay/cell flocs, forming a net-like structure that entrains cells deeper in the water column through sweep capture [32].

One specific formulation of PAC-modified kaolinite clay is called Modified Clay-II (MCII). Previous studies evaluated the effectiveness of MCII in removing *K. brevis* and associated brevetoxins from seawater [33,34]. These studies monitored changes in key water quality parameters including pH, temperature, salinity, and dissolved oxygen within both the water column and the settled floc. In addition, potential lethal and sublethal effects on several benthic marine organisms were assessed. Results demonstrated that MCII was effective in removing *K. brevis* cells from surface waters, though most of the toxins, particularly derivative toxin congeners, still remained in the water column. It was presumed that the fragile *K. brevis* cells potentially ruptured and released toxin during the flocculation and sedimentation process. Notably, MCII treatment had no significant impact on co-occurring marine species, with no observed adverse effects on behavior, respiration, or mortality.

Although having been shown initially to be effective at removing *K. brevis* cells from affected seawaters, an important unresolved question is whether there is any potential impact of the settled MCII clay floc on benthic animal species. Specifically, we aimed to determine whether brevetoxins captured in the clay floc are available to these organisms over time, and whether prolonged exposure to the aluminum-containing (PAC) MCII clay/floc could result in metal toxicosis in species that remain in contact for extended periods after a treatment has taken place. To address these concerns, we conducted a small-scale mesocosm study using 80 L cylindrical tanks inoculated with *K. brevis* and treated with MCII. The primary objectives of this study were to (1) confirm previous findings regarding the effectiveness of MCII as a flocculation agent for *K. brevis* cell removal; (2) assess the potential release of BTX from settled floc back into the water column and sediment; and (3) quantify BTX and trace metal uptake in the tissues of clams introduced into the floc post-application to evaluate bioavailability and potential toxicological effects. We anticipate that these findings will serve as a foundation for future research on MCII flocculation and its effects on marine benthic species, while also advancing the understanding of brevetoxin dynamics following capture by PAC-modified clays. These results provide an important step toward advancing research on MCII flocculation and its use as a HAB mitigation strategy.

## 2. Materials and Methods

### 2.1. Mercenaria mercenaria Husbandry

Hard clams (*M. mercenaria*) were purchased from Southern Cross Sea Farms, Cedar Key, FL. Red tide blooms are infrequent at this harvest site, and animals were confirmed to have zero background brevetoxin accumulation prior to the experiment. Animals were transported in a cooler, scrubbed of mud and debris, and acclimated in a raceway prior to beginning exposure. During acclimation, water was provided in a flow-through system and salinity was increased by 2 ppt every day to a final salinity of 30 ppt. Shellfish were fed *Thalassiosira weissflogii* ((Grunow) G. Fryxell & Hasle) daily during maintenance and throughout the experiment.

### 2.2. Karenia brevis Culturing

*Karenia brevis* (Mote New Pass Clone/CCMP 2228) was cultured in modified L1 media (without SiO_4_) at 24 °C, salinity 34-36 ppt, and a 12 h L:D cycle at 50–60 μmol/m^2^/s to a stock density of 15.0–25.0 × 10^6^ cells/L. Cultures were expanded by weekly transfers of 1:5 stock:media, using a sterile technique, until sufficient cell numbers were obtained. Stock culture was diluted in filtered seawater to a target density of 1 × 10^6^ cells/L as described below. 

### 2.3. Experimental Design and Sampling Methods

This experiment was carried out at the indoor mesocosm facility of the Florida Red Tide Mitigation and Technology Development Initiative (RTMTDI) at Mote Marine Laboratory in Sarasota, FL, USA during the week of 7 April 2025. Sarasota Bay, a local estuary that feeds into the Gulf of Mexico, is relatively shallow with an average depth of about 1.75 m [35]. This region is regularly affected by *K. brevis* blooms, and the experimental design was intended to simulate conditions under which future deployment of MCII might occur.

**Figure 2:**
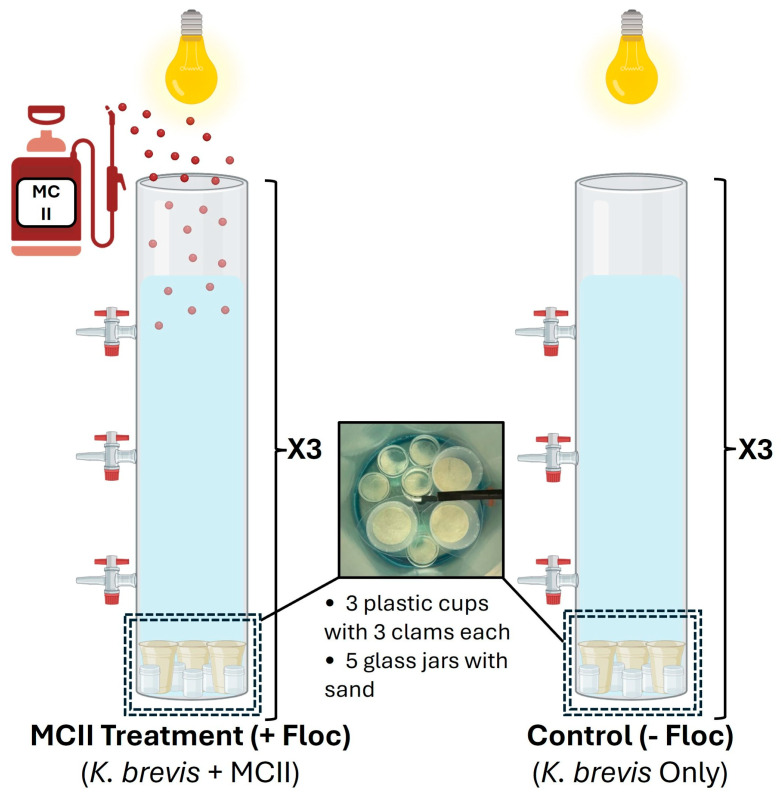
Experimental setup of 80 L tanks to assess the effects of MCII. Each setup included three replicate columns and illuminated with overhead lighting. The treatment group (**left**) was exposed to both *K. brevis* and MCII (0.2 g/L), while the control group (**right**) was exposed to *K. brevis* only. The bottom of each column contained 3 plastic cups (each with 3 clams) and 5 glass jars filled with sand to simulate sediment conditions.

In order to simulate a vertical cutout of the Sarasota Bay water column, six cylindrical fiberglass tanks (height 1.22 m, diameter 0.31 m), each with a capacity of 80 L, were assembled as shown in Figure 2. Each tank was fitted with three taps positioned at specific depths to allow representative sampling from the surface, middle, and bottom of the simulated water column. At the base of each tank, three plastic cups containing approximately 150 mL of sand were placed to hold clams and collect floc after treatment has taken place. Additionally, five glass jars filled with approximately 20 g of sand were included in each tank to collect settled floc for sediment toxin analysis. Overhead lights were installed above each tank to ensure consistent illumination for the added cellular culture. The experiment was conducted at 30 ppt, within the range of *K. brevis* salinity tolerance and the natural conditions of Cedar Key clams. Any discrepancy between the measured salinities of *M. mercenaria* and *K. brevis* during the growth and acclimation phase is not expected to affect the experimental outcomes. Tanks were carefully filled with seawater to avoid disturbing the sand and then inoculated with *Karenia brevis* culture, reaching a final concentration of approximately 6 × 10^5^ cells per liter. The final volume of 80 L per tank corresponded to roughly 48 million cells. After inoculation, the culture was allowed to acclimate for 24 h.

**Figure 3:**
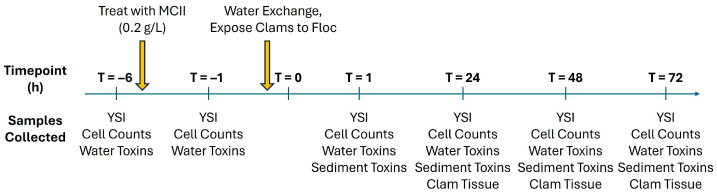
Sampling timeline for YSI water quality, cell counts, water toxins, sediment toxins and clam tissue toxins. Yellow arrows indicate MCII treatment time point and time at which water was swapped in each tank followed by addition of clams.

Figure 3 illustrates the timeline of the experiment including clay treatment, water exchange, and samplings. Following the *K. brevis* culture acclimation period, ProDSS YSI (YSI Inc., Yellow Springs, OH, USA) water quality measurements (pH, oxidation-reduction potential, salinity, dissolved oxygen, and temperature) were taken by lowering the probe halfway through the tank (0.61 m), and integrated water samples were collected from the three taps to establish baseline conditions and cell concentrations prior to the MCII clay treatment (T = −6). Throughout the experiment, all water samples were collected by first opening the taps and allowing water to flow for 3 s to clear the ports. From each tank, approximately 20 mL of water was collected at each designated depth to ensure representative sampling of the entire water column. The three representative samples were then combined into a single pooled sample for extraction and analysis by UHPLC-MS (total 50 mL). From this pooled sample, roughly 10 mL of water was pipetted into a clean glass vial containing 1% Utermöhl’s solution for subsequent cell counting analysis.

Immediately following pre-treatment water sampling (T = −6), a slurry consisting of 16 g of MCII mixed with 300 mL seawater was prepared and transferred into handheld garden sprayers. Tanks were then randomly designated as either Control (No Floc) or MCII Treatment (Floc Present). If a control tank was located adjacent to a treatment tank, the top of the control tank was covered with cardboard during clay application to prevent any overspray from contaminating control tank water. The sprayer was manually pressurized, shaken, and then used to apply the slurry into the designated tanks. To maintain a consistent application, the sprayer was periodically re-pressurized and shaken throughout the process. Each MCII treatment tank received approximately 300 mL of slurry, corresponding to an application concentration of 0.2 g/L. Clay was allowed to flocculate and settle for 5 h. After the 5 h MCII clay flocculation and settling period was completed (T = −1), ProDSS YSI water quality measurements (pH, oxidation-reduction potential, salinity, dissolved oxygen, and temperature) were taken in addition to collection of water samples to measure the effectiveness of MCII clay treatment on *K. brevis* cell and toxin removal.

After the T = −1 measurements were completed, we wanted to turn our focus towards the settled MCII floc. To accurately study the effects of only the settled floc after treatment and whether captured toxins within the floc were accessible to benthic species, water from all six tanks was very carefully removed and exchanged with fresh seawater. This was performed to isolate the floc away from treated waters and ensure that measured toxin concentration changes we observed could be directly attributed to the presence or absence of the floc itself and not *K. brevis* cells that might remain in the water after treatment. Water exchange in control tanks was performed to mimic the conditions of treatment tanks, minus the presence of settled floc. Water exchange was performed in conjunction with the addition of three *M. mercenaria* clams into each plastic cup containing approximately 150 mL of sand (9 total clams per tank). This species of clam is a staple in Florida shellfish aquaculture and common in the coastal regions. Fresh seawater was then added to each tank very slowly to ensure sand and clay floc in each cup/jar was not disturbed. Completion of water exchange marked the start of T = 0, indicating the beginning of *M. mercenaria* clam exposure to MCII floc after initial flocculation and sedimentation had already taken place. Subsequent water samples and ProDSS YSI quality measurements were taken from each tank as described above at predetermined hourly time points T = 1-, 24-, 48-, and 72 h post MCII floc exposure to clams (Figure 3).

Sediment sampling occurred at time points T = 1-, 24-, 48-, and 72 h post MCII floc exposure (Figure 3). During sampling periods, one glass jar containing sediment was randomly selected from each tank and carefully removed so as not to disturb the contained sand and floc. Upon surfacing, jars were capped to prevent sample loss and extracted immediately.

*Mercenaria mercenaria* tissue sampling occurred at time points T = 24-, 48-, and 72 h post MCII floc exposure (Figure 3). For clam tissue sampling, one plastic cup containing three clams was randomly selected from each experimental tank, removed and culled by incubating at −20 °C overnight. After, all three clams from a single plastic cup were then shucked, homogenized and pooled together using a hand-held blender. Individual clam tissue originating from the same plastic cup were pooled together to obtain a representative sample of each individual treatment tank. These samples were stored at −20 °C until extraction.

### 2.4. Extraction of Water, Sediment, and Tissue Brevetoxins

For UHPLC-MS analysis of water samples, 50 mL of test water was extracted using a Promochrom SPE-03 system (Promochrom Technologies, Richmond, BC, USA) equipped with Phenomenex (Torrance, CA, USA) Strata C18-E SPE cartridges (55 µm, 70 Å, 200 mg/3 mL). Cartridges were conditioned with 5 mL of 100% methanol at a flow rate of 5 mL/min, followed by equilibration with 5 mL of deionized (DI) water at the same flow rate. Samples were then loaded onto the cartridges and passed through at 10 mL/min. This was followed by a 5 mL rinse with reverse osmosis (RO) water at 5 mL/min. Cartridges were dried for 6 min using ultra-high purity (UHP) nitrogen gas, then eluted with 5 mL of 100% methanol at a flow rate of 5 mL/min. The cartridge beds were purged to ensure full recovery of the elution solvent. Eluates were evaporated to dryness under UHP nitrogen while heated in a 40 °C water bath for approximately one hour. Dried samples were then reconstituted in 3 mL of methanol for UHPLC-MS analysis. At each sampling time point, one blank and one standard recovery control were processed, along with three analytical duplicates (approximately one set for every nine samples).

For the extraction of brevetoxins in sediment, a Buchner funnel was put under vacuum and equipped with a Whatman 70 mm glass microfibre filter (GF/F). Clay was rinsed out of the jar and collected on the filter. After, sand was rinsed out of the jar and collected on a separate filter. Once sediment samples were filtered, 0.5 g of floc and 2.0 g of sand were weighed out and pooled together into a 50 mL plastic centrifuge tube. These samples were then extracted by a modified QuEChERS method. In short, 20 mL of RO water and 15 mL of acetonitrile (Fisher Chemical, Waltham, MA, USA) were added to the centrifuge tube and hand-shaken for 1 min before adding in the full QuEChERS packet (Waters, DisQuE, Milford, MA, USA) and hand-shaken for another minute. Samples were sonicated for 20 min before being placed on a rocking table at approximately 210 rpm for 25 min. After removal from the rocking table, the samples were centrifuged for 8 min at 4200 rpm. 10 mL of the acetonitrile layer was aliquoted into 15 mL centrifuge tubes and evaporated to dryness under UHP nitrogen while heated in a 40 °C water bath for approximately two hours. Dried samples were then reconstituted in 2 mL of LC/MS grade methanol before filtering through 0.22 μm PTFE filters (Thermo Scientific, Waltham, MA, USA) for UHPLC-MS analysis.

For the extraction of clam tissue toxins, approximately 1 g of homogenized tissue was taken from each pooled sample, weighed and transferred to a vial containing 9 mL of 80:20 methanol:water. Samples were then vortexed for 1 min, then sonicated for 5 min, and vortexed again for 1 min. Finally, samples were centrifuged at 3000 rpm for 5 min to pellet the extracted tissue. The supernatant was decanted into separate 15 mL centrifuge tubes and extracts were evaporated to dryness under UHP nitrogen while heated in a 40 °C water bath for approximately two hours. Dried samples were then reconstituted in 1 mL of methanol and filtered using a 0.22 μm PTFE filter. At each sampling time point, one blank and one standard recovery control were processed, along with three analytical duplicates (approximately one set for every nine samples).

### 2.5. Cell Counts and Quantification of Brevetoxins in Water, Sediment and Tissue Samples

To quantify *K. brevis* cells per tank, 10 mL water samples were immediately preserved with approximately 1% Utermöhl’s solution in glass scintillation vials and stored at ambient temperature (22 °C) until analysis. A 1 mL aliquot from each sample was examined using a gridded Sedgewick-Rafter counting chamber under an Accu-Scope (Commack, NY, USA) EXI-310 light microscope at 20× magnification. At least 100 cells, or a volume of 100 µL, were counted per sample. For samples with very low cell concentrations, the entire chamber was analyzed.

Brevetoxin analysis was conducted using reversed-phase liquid chromatography coupled with a triple quadrupole (TQ) mass spectrometer equipped with an electrospray ionization interface (LC-ESI-MS/MS). The system consisted of a Thermo Fisher Scientific TSQ Quantis mass spectrometer (Thermo Fisher Scientific Inc., Waltham, MA, USA, NYSE: TMO) integrated with a Vanquish LC platform, which included a binary pump, autosampler, and temperature-controlled column compartment. Chromatographic separation was performed using a Hypersil Gold Vanquish Aq UHPLC C_18_ reversed-phase, polar end-capped column (1.9 µm particle size, 100 mm × 2.1 mm I.D.), also from Thermo Fisher Scientific Inc., USA. The mobile phase comprised water with 0.1% formic acid (solvent A) and acetonitrile with 0.1% formic acid (solvent B). Gradient elution began at 50:50 (A:B, *v*/*v*) for 1 min, followed by a linear increase to 95% B over 9 min. This composition then returned to the initial conditions over 1 min and held for 4 min. The total run time was 15 min, with a constant flow rate of 200 µL/min and an injection volume of 5 µL. The column temperature was maintained at 30 °C. Quantification was performed using multiple reaction monitoring (MRM), with parameters optimized via high-flow infusion. Ionization was achieved in positive mode using nitrogen as both the nebulizer and drying gas. The ESI source was operated at a spray voltage of 4745 V, with a vaporizer temperature of 75 °C, a transfer temperature of 350 °C, and sheath, auxiliary, and sweep gas pressures set to 25, 16.2, and 0 (arbitrary units), respectively. These conditions were applied throughout the analysis. Absolute quantification was conducted for BTX-1, -2, and -3, as well as BTX-2-carboxylic acid (BTX-B5) using certified standards (MARBIONC, University of North Carolina, Wilmington, NC). Method validation included recovery experiments, in which known amounts of brevetoxins were spiked into water, sediment, and tissue matrices followed by extraction. Relative quantification was used for brevetoxins B2, A2, and their conjugated derivatives (SdoxB2 and SdoxA2), for which no analytical standards were available (Tables S7–S10).

### 2.6. Metals Analysis of Clam Tissue

Clam homogenate samples for trace metal analysis were stored in a −20 °C freezer prior to digestion. All sample handling was performed in a class 100 clean room. Samples were transferred into pre-tared and acid-cleaned 15 mL PFA vials (Savillex, Eden Prairie, MN, USA), weighed, and dried at 60 °C for 42 h to constant weight. The dried samples were digested alongside reference materials (DORM-5 National Research Council Canada; BCR-414 European Commission Joint Research Center) following an established procedure [36]. Briefly, 3 mL of concentrated nitric acid (HNO_3_; Optima, Fisher, Schwäbisch Hall, Germany) and 0.375 mL concentrated hydrogen peroxide (H_2_O_2_; Optima, Fisher) were added to each vial; the vial was then capped and gently inverted. The caps were then removed, and the vials were allowed to react at room temperature for 20 min. After 20 min, the vials were recapped loosely and placed on a hotplate at 125 °C for 8 h. Upon exposure to heat we observed that some of the vials began to bubble over, which may have contributed to a small loss of sample material; we temporarily removed the vials from the hotplate to allow a few more minutes of reaction at room temperature before resuming the digestion on the hotplate. Following 8 h at 125 °C, vial caps were removed, and the hot plate temperature was reduced to 60 °C for 5 h to evaporate remaining liquid. The residue was dissolved in 10 mL of 10% HNO_3_ (Optima, Fisher). The reference material results from the digestion compared well with previous studies and we expect minimal loss of material during the digestion. Out of an abundance of caution, however, we also report results in both mg metal/kg dry mass as well as metal:phosphorus ratios (mmol metal/mol P), using P as a proxy to normalize biomass concentrations digested in each sample.

Samples were analyzed using inductively coupled mass spectrometry (ICP-MS; iCAP, ThermoFisher) in collision cell mode, using an ESI SC4-DX autosampler. Prior to analysis samples were further diluted at 1:10 and 1:100 ratios in acid-cleaned falcon tubes using 5% HNO_3_ (Optima, Fisher), then spiked with 1 µm/kg indium (In) as an internal standard. Twelve calibration standards (range of 10 ng/kg^−1^ mg/kg) were prepared in the same 5% Optima HNO_3_-1 µm/kg In matrix from SPEX stock solutions for target analytes, aluminum (Al), phosphorous (P), scandium (Sc), vanadium (V), chromium (Cr), manganese (Mn), iron (Fe), cobalt (Co), nickel (Ni), copper (Cu), zinc (Zn), arsenic (As), strontium (Sr), yttrium (Y), molybdenum (Mo), cadmium (Cd), barium (Ba), lutetium (Lu), and lead (Pb). Instrument response for each analyte was normalized to the In internal standard to correct for instrument variability, and the target analyte concentrations in each sample quantified using the standard curves.

### 2.7. Statistical Analysis

For the entirety of the experiment, statistical comparisons between groups were conducted using a two-tailed, two-sample unequal variance Welch’s *t*-test, a modified *t*-test that adjusts degrees of freedom to account for unequal variances [37]. Highlighted in select figures, the level of significance is indicated for water quality measurements, *K. brevis* cellular concentrations and *M. mercenaria* tissue aluminum quantities (* *t* < 0.01, *** *t* < 0.0001). Additional statistical data for the determination of aluminum concentrations in tissue can be found within the Supplemental Data provided (Tables S1–S6).

## 3. Results

### 3.1. Water Quality Measurements

**Figure 4:**
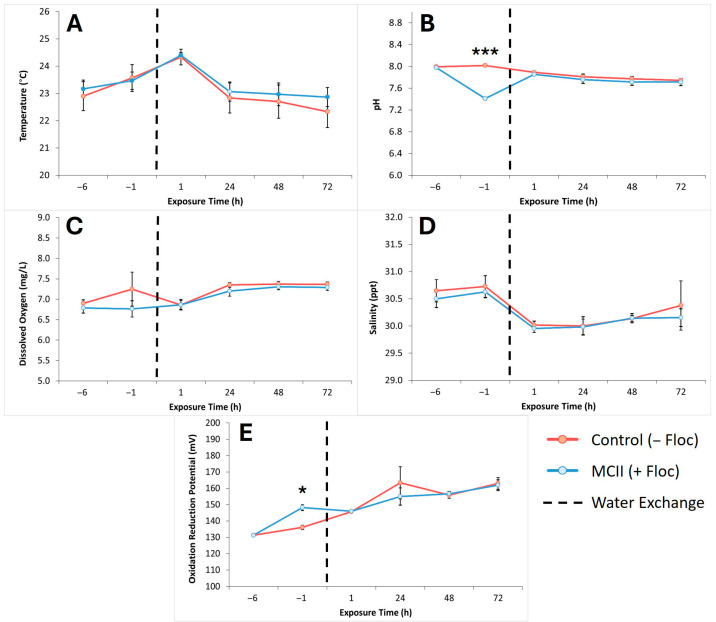
Mean ± standard deviation of YSI water quality measurements of (**A**) temperature (°C), (**B**) pH, (**C**) dissolved oxygen (mg/L), (**D**) salinity (ppt), and (**E**) oxidation reduction potential (mV) taken over time from control (−Floc) and MCII treatment (+Floc) tanks (3 tanks averaged per control and treatment). Red line represents control (−Floc). Blue line represents MCII treatment (+Floc). Vertical black dashed line represents time at which water was exchanged in each tank and clams were added. The level of significance is indicated for cellular concentrations (* *t* < 0.01, *** *t* < 0.0001) using Welch’s *t*-test, two-tailed, two-sample unequal variance.

At each sampling time point, water quality parameters including temperature, pH, dissolved oxygen, salinity, and oxidation-reduction potential (ORP) were measured in all six tanks to assess the effects of the MCII treatment (Figure 4A–E). For each parameter, the average values from the three control tanks (−floc) and the three MCII treatment tanks (+floc) were used for data visualization. Water temperatures remained consistent across all tanks throughout the study, with only slight increases observed at T = 1 (Figure 4A). Differences in pH between treatment and control groups were noted, with a slight decrease in pH observed in the treatment group at T = −1 (Figure 4B). Dissolved oxygen levels were analyzed to identify any differences between the MCII treatment and control groups; however, no significant variation was detected (Figure 4C). Similarly, changes in salinity were minimal. Both treatment and control tanks exhibited a slight decline in salinity when tank water was replaced (Figure 4D). Oxidation-reduction potential (ORP) was also monitored across all tanks. During the first five hours of treatment, ORP in the MCII group increased by 8.9% compared to the control. One hour after water exchange and clams were added (T = 1), ORP levels in both groups equalized with subsequent increases occurring at comparable rates (Figure 4E).

### 3.2. Water, Cell Counts, Sediment, and Tissue Toxin Analysis

**Figure 5:**
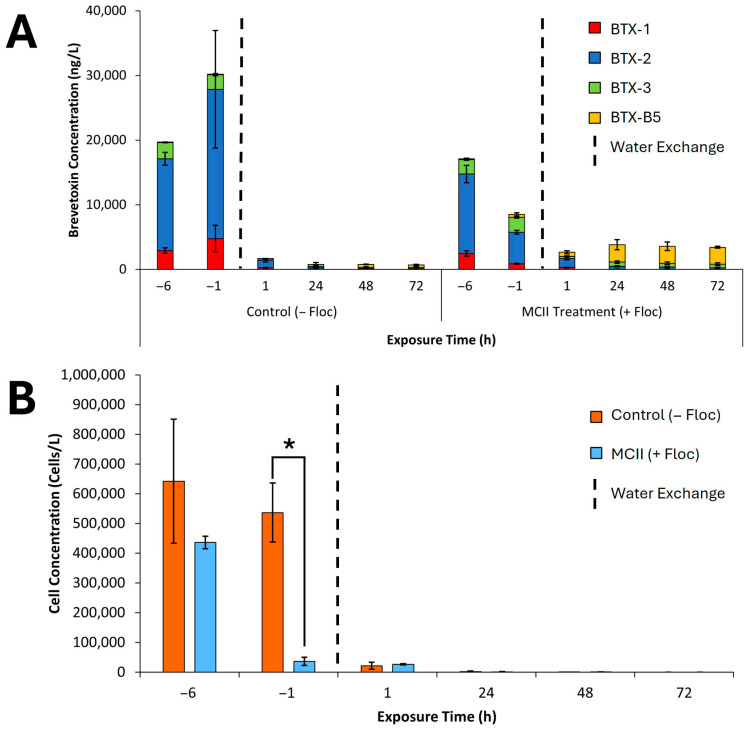
(**A**) Mean ± standard deviation of total brevetoxin concentrations (ng/L) measured in water over time in Control (−Floc) and MCII Treatment (+floc) tanks (*n* = 3 tanks per group). Vertical black dashed lines indicate the time points when water was exchanged, and clams were introduced. Total brevetoxin levels represent the sum of individual toxin congeners detected in each tank. (**B**) Mean ± standard deviation of *K. brevis* cell concentrations (cells/L) over time in Control (−Floc) and MCII Treatment (+floc) tanks (*n* = 3 tanks per group). Vertical black dashed lines denote the timing of water exchange and clam addition. The level of significance is indicated for cellular concentrations (* *t* < 0.01) using Welch’s *t*-test, two-tailed, two-sample unequal variance.

The concentration of brevetoxins and *K. brevis* cells in water was measured over a 78 h period in both control and MCII-treated conditions (Figure 5A,B). In both control and treatment water samples prior to MCII application (T = −6), initial toxin concentrations exhibited high total brevetoxin, reaching nearly 20,000 ng/L. The predominant toxin at these time points was BTX-2, followed by BTX-1, BTX-3, and BTX-B5 (Figure 5A). Likewise, prior to MCII treatment, total detected cells in both control and MCII tanks were comparable, averaging around 539,000 cells/L between all tanks (Figure 5B). Following MCII treatment and a five-hour flocculation period (T = −1), total brevetoxin concentrations in treated water decreased by nearly 50%, whereas control water samples displayed a significant increase in total toxin level, reaching roughly 30,200 ng/L (Figure 5A). Of note, comparison of measured water and sediment toxin concentrations revealed that only a small portion of the captured brevetoxin in MCII treated water was caught in the glass sample jars during the five-hour flocculation period. The remaining flocculated brevetoxin potentially could have been captured in the other plastic sample cups housing clams or perhaps evaded capture altogether and settled onto the bottom of the tanks. Comparison of total cell concentrations of control versus MCII-treated water revealed MCII treatment substantially reduced *K. brevis* concentrations by nearly 91%, demonstrating a highly effective flocculation event (Figure 5B).

As expected, one hour after the water in all six tanks was exchanged and clams were introduced (T = 1), a significant reduction in total brevetoxin concentration was observed in both control (−floc) and MCII treated (+floc) water samples (Figure 5A). Notably, MCII treated (+floc) water samples displayed emergence of BTX-B5, presumably from the sedimented floc. After water exchange, total detected cells in both groups were relatively low at approximately 25,000 cells/L (Figure 5B). This trend continued with further decreases at T = 24, 48, and 72 h, where total brevetoxin levels and cell counts remained consistently reduced. Notably, the residual brevetoxin composition in MCII-treated water was primarily BTX-B5 after 24 h, with BTX-2 and BTX-1 becoming increasingly negligible.

**Figure 6:**
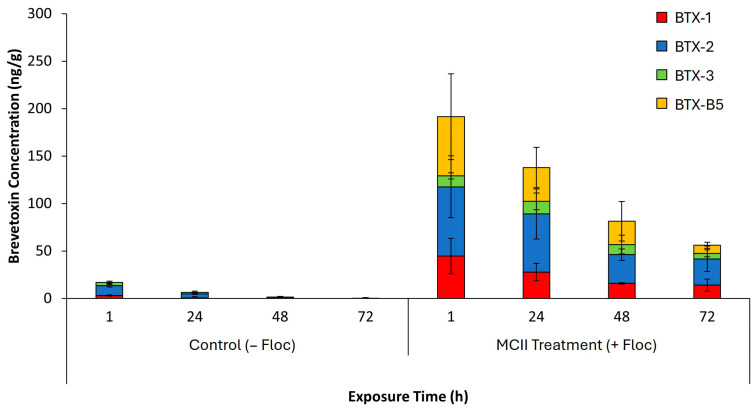
Mean ± standard deviation of total brevetoxin concentrations (ng/g) measured in sediment over time in Control (−Floc) and MCII Treatment (+Floc) tanks after water exchanges (*n* = 3 tanks per group). Total brevetoxin levels represent the sum of individual toxin congeners detected in each tank.

Brevetoxin concentrations in sediment were measured over a 72 h exposure period in both control (−floc) and MCII-treated (+floc) conditions (Figure 6). In control (−floc) sediments, brevetoxin concentrations remained low across all time points (≤25 ng/g), with BTX-2 as the dominant congener. By 72 h, toxin levels were nearly undetectable. In contrast, MCII-treated (+floc) sediments showed significantly higher brevetoxin content, initially measured at 191 ng/g and gradually decreasing to approximately 47 ng/g after 72 h. All congeners were present, with BTX-1, -2 and -B5 contributing most to total levels.

**Figure 7:**
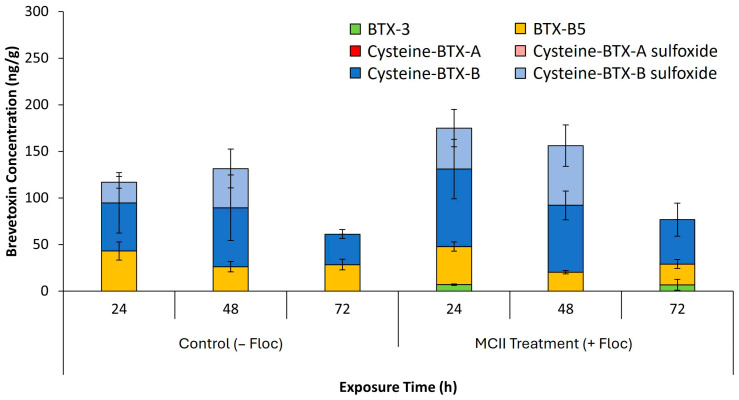
Mean ± standard deviation of total brevetoxin concentrations (ng/g) measured in clam tissue over time in Control (−Floc) and MCII Treatment (+Floc) tanks after water exchanges (*n* = 3 tanks per group). Total brevetoxin levels represent the sum of individual toxin congeners detected in each tank.

Brevetoxin concentrations in *Mercenaria mercenaria* clam tissue were assessed in both control (−floc) and MCII-treated (+floc) groups (Figure 7). In control (−floc) tissues, total brevetoxin levels peaked after 48 h exposure (T = 48) with approximately 131 ng/g detected. Cysteine-BTX-B and Cysteine-BTX-B sulfoxide were observed to be the dominant congeners. By 72 h, concentrations decreased to roughly 61 ng/g. Similarly, MCII-treated (+floc) tissues showed peak brevetoxin accumulation at 24 h exposure, with total average concentration of approximately 175 ng/g, comparable to control tissues. Cysteine-BTX-B remained the predominant congener, while BTX-3 made up a smaller proportion. By 72 h, toxin levels declined to about 77 ng/g, mirroring the control (−floc) trend.

### 3.3. Metals Analysis

**Figure 8:**
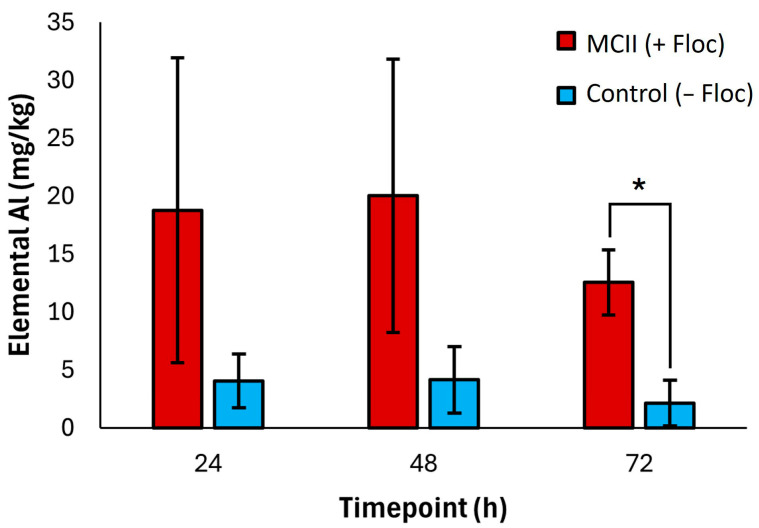
Mean ± standard deviation of trace aluminum (Al) concentrations within tissue of MCII Treatment (+Floc) and Control (−Floc) groups (*n* = 3 clams per group). Tissue samples were collected at 24-, 48-, and 72 h post treatment and analyzed by ICP-MS. Concentrations of target analyte are expressed as milligrams of target analyte per kilogram of clam tissue (mg/kg). The level of significance is indicated for aluminum concentration (* *t* < 0.01) using Welch’s *t*-test, two-tailed, two-sample unequal variance.

Eighteen elements were quantified in clam tissues using inductively coupled plasma mass spectrometry (ICP-MS). Clam tissues from MCII treated (+floc) and control (−floc) tanks were collected at 24-, 48-, and 72 h exposure. Concentrations of each analyte were expressed as milligrams of target element per kilogram of clam tissue (mg/kg) and as normalized to biomass in units of mmol element per mol phosphorus. Across all time points, MCII treated (+floc) clams exhibited consistently higher aluminum (Al) levels compared to controls (−floc). Specifically, Al concentrations in MCII treated (+floc) clams ranged from approximately 12.6–20.0 mg/kg, whereas control clam (−floc) tissues remained at or below 4.2 mg/kg (Figure 8). In contrast, no significant differences were observed between MCII treated and control groups for phosphorus (P), scandium (Sc), vanadium (V), chromium (Cr), manganese (Mn), iron (Fe), cobalt (Co), nickel (Ni), copper (Cu), zinc (Zn), arsenic (As), strontium (Sr), yttrium (Y), molybdenum (Mo), cadmium (Cd), barium (Ba), lutetium (Lu), or lead (Pb) (Figure S1).

## 4. Discussion

The goals of this study were to replicate previous findings regarding the effectiveness of MCII as a flocculation agent for *K. brevis* cell removal and assess any potential effects of the generated floc to benthic organisms, specifically brevetoxin bioavailability from settled floc and trace metal uptake in the tissues of clams. Overall, the data demonstrate that MCII effectively reduces waterborne *K. brevis* cells while enhancing brevetoxin partitioning into sediment without significantly affecting water quality or toxin accumulation in clam tissues.

Closer analysis of water quality parameters indicated that MCII treatment and prolonged floc exposure had minimal impact on temperature, dissolved oxygen, or salinity (Figure 4A,C,D). During the first five hours of the experiment, temperatures in both the treatment and control groups increased by approximately 0.5 °C, followed by a gradual return to baseline levels over the subsequent 72 h. We surmise that variations in temperature reflect ambient temperature changes caused by the surrounding environment and were unrelated to MCII treatment. A comparison of water pH (Figure 4B) during the flocculation phase of the MCII treatment showed a decrease in the treatment groups, from 8.0 to 7.4. As previously mentioned, this outcome was anticipated, as MCII treatment generates cationic species in the water [32], which in turn lowers the pH. Furthermore, this was also observed in previous mesocosm studies investigating MCII as a mitigation strategy [34]. Following water exchange, pH values returned to their initial levels and remained stable thereafter. Both dissolved oxygen and salinity remained within normal ranges throughout the experiment, with a slight increase in dissolved oxygen observed in the control tanks at T = −1, rising from 6.9 to 7.2 mg/L.

Specifically following clay application and flocculation, a marked increase in oxidation-reduction potential (ORP) was observed in MCII treated samples compared to controls at T = −1) (Figure 4E). Compared to control (−floc) tanks at that time point, MCII treatment (+floc) resulted in an increase in ORP of approximately 12 mV before water exchange. Elevated ORP values typically reflect the presence of strong oxidizing agents and are often associated with improved water quality and enhanced contaminant-neutralizing capacity [38,39]. This increase aligns with the proposed mechanism of MCII, which involves the hydrolysis of embedded aluminum salts upon application. This process generates positively charged aluminum species, such as Al(OH)^2+^ and Al(OH)_2_^+^, contributing to increased redox activity [32]. The greater abundance of these cationic aluminum species in MCII treated water supports the occurrence of oxidation processes, indicating a more chemically reactive environment relative to control tank conditions. It was noted that overall ORP values increased gradually over time in all tanks throughout the course of the experiment, including the controls. We speculate that the addition of clams, their daily *Thalassiosira weissflogii* feed and normal metabolic processes could have had a positive influence, but this remains to be confirmed. Nonetheless, after water exchange was performed in all tanks, there was no significant difference in measured ORP values between MCII treated water compared to controls. Taken altogether, this data demonstrates that the use of, and prolonged presence of MCII floc has minimal effect on water quality.

Further analysis of waterborne brevetoxins and *Karenia brevis* cell concentrations demonstrated a significant effect of MCII treatment (Figure 5A,B). Following application and flocculation (T = −6 and −1), MCII treated (+floc) water showed an approximate 50% reduction in brevetoxin concentrations from 17,131 ng/L to 8532 ng/L, while control (−floc) samples exhibited a 1.5-fold increase over the same period (19,705 ng/L to 30,192 ng/L). In both groups, BTX-2 remained the dominant brevetoxin congener. Notably, MCII treatment led to a substantial decline in *K. brevis* cell densities, achieving nearly a 91% reduction after just five hours of flocculation compared to controls. These results align with other studies investigating MCII as a mitigation strategy and highlight the ability of PAC-modified clays to remove algal colonies from water [23,27,34]. As expected, water replacement in control tanks resulted in minimal detectable brevetoxins; however, residual levels may be attributed to toxin adhering to tank surfaces or additional toxin production from residual *K. brevis* cells. Likewise, water replacement in both control and MCII tanks resulted in minimal to no detected cellular presence in sampled water.

Notably, following water replacement in MCII treated (+floc) tanks, brevetoxin concentrations remained elevated relative to controls (−floc). This suggests the possibility that brevetoxins previously captured by MCII could leach out into the water over time, or perhaps living cells present in the floc could subsequently escape, further introducing toxins into the water column. Indeed, unpublished studies by V. Lovko et al. utilizing vital stains of floc samples after MCII treatment revealed some living *K. brevis* cells in the MCII floc. Moreover, the congener profile shifted, with BTX-B5 emerging as the dominant species. As BTX-B5 is the oxidized form of BTX-2, this redistribution suggests that the highly oxidizing conditions induced by MCII are promoting the conversion of BTX-2 to BTX-B5, which was the predominant congener prior to the water exchange. The presence of hard clams may also contribute to water toxins, as they detoxify ingested brevetoxins and excrete modified congeners, including BTX-B5. This conversion could be viewed as beneficial, as the lethal dose 50% (LD_50_) of mice administered BTX-2 intraperitoneal (i.p.) is approximately 0.2 mg/kg, whereas the LD_50_ of BTX-B5 is in the range of 0.3–0.5 mg/kg, making it the less toxic congener [40].

Analysis of sediment toxin profiles revealed a substantial difference between control (−floc) and MCII-treated (+floc) conditions (Figure 6). In control sediments, brevetoxin concentrations remained low across all time points, with levels ranging from 6 to 17 ng/g and becoming nearly undetectable by 72 h. This is as expected given that only a small number of cells or settled material would have been present in the control tanks after water replacement. BTX-2 was the predominant congener in these samples, with minimal contributions from BTX-1, BTX-3, and BTX-B5. In contrast, MCII treated (+floc) sediments exhibited a marked increase in total brevetoxin accumulation, initially measured at 191 ng/g and gradually declined to 56 ng/g by 72 h. All four congeners were present in MCII samples, with BTX-2 and BTX-B5 contributing most significantly. Brevetoxins analyzed in sediments potentially include toxins adhered to clay particles, toxins in flocculated *K. brevis* cells, toxin contributions from interstitial water in the floc, and toxins in clam feces or pseudofeces. Aligning with measured water toxin samples, the elevated levels of BTX-B5, an oxidized derivative of BTX-2, suggest that the oxidizing environment induced by MCII promotes transformation of brevetoxins in addition to enhancing their sedimentation.

These and others sediment analysis results reveal a potential drawback of using PAC-modified clays to flocculate toxin-producing HABs in that a significant portion of the initial toxins in the water column were transported to bottom sediments in the floc [33,34]. Future users of PAC-modified clays in impacted water bodies should take this into consideration before application occurs in order to assess the ramifications of mitigation on the local environment. As discussed by Devillier et al. ([33]), the one-time impact of sedimented toxins following clay treatment may be small relative to the long-term impact of an untreated bloom that continues to grow and persist for weeks or months.

Tissue analysis of *Mercenaria mercenaria* clams exposed to control (−floc) and MCII treated (+floc) environments from 24 to 72 h is presented in Figure 7. At all sampling time points, clams in the MCII treated (+floc) environment exhibited slightly higher brevetoxin concentrations compared to those in control (−floc) conditions. Although the differences were not statistically significant, the trend suggests that flocculation may increase benthic exposure to brevetoxins by concentrating *K. brevis* cells and their associated toxins in the lower water column, however further studies are needed to confirm. An important observation during the study was the increased accumulation of pseudofeces in plastic cups housing clams within MCII treated (+floc) tanks compared to controls (−floc). Bivalve mollusks are known to selectively reject and expel non-nutritive suspended particles through pseudofeces production [41]. We hypothesize that clams in the treatment tanks may have actively rejected clay particulates introduced by MCII during feeding, potentially reducing the amount of brevetoxins accumulated in their tissues. This selective feeding behavior could spare *M. mercenaria* from brevetoxin accumulation via floc. Furthermore, flocculation of cells may ultimately reduce brevetoxin tissue accumulation in comparison to exposure to an untreated bloom. To better understand these dynamics, future studies should include a broader range of benthic species with differing feeding strategies. We hope this provides a clearer understanding of how flocculating HABs, which effectively concentrate the bloom onto the seafloor, impacts benthic species moving into and out of the treatment zone. Importantly, no clam mortalities attributable to flocculation-induced brevetoxicosis were observed in this study, consistent with previous findings [33,34].

Quantification of eighteen metal elements in clam tissue was conducted to evaluate whether MCII treatment (+floc) led to increased accumulation relative to controls (−floc). This analysis was performed to assess potential toxicological implications for resident benthic species. MCII is a polyaluminum chloride (PAC)–modified clay, in which aluminum (Al) serves as a key structural component to enhance algal flocculation. In its polymeric form, aluminum species generate cationic surface patches that promote bridging between algal cells and suspended clay particles. This interaction facilitates the formation of larger, networked flocs that settle rapidly from the water column, thereby promoting the sweep removal of *K. brevis* from the treated environment [32]. Notably, across all time points, clams exposed to MCII treatment (+floc) accumulated aluminum at concentrations averaging 5.1-fold higher than those observed in controls (−floc) (Figure 8), whereas accumulation of other measured trace metals was not significant between treatment and control groups (Figure S1). Previous studies examining the acute and chronic toxicity of aluminum in freshwater unionid mussels and amphipods reported acute 50% effect concentrations (EC_50_) for survival exceeding 6200 µg total Al/L, indicating that both species exhibit low sensitivity to dissolved aluminum under acute exposure conditions [42]. While direct correlations remain uncertain, we hypothesize that benthic species in marine environments may exhibit similar insensitivity to aluminum. This is supported by the absence of clam mortality in the present study, as well as previous investigations demonstrating that MCII treatment did not significantly affect co-occurring marine species, with no observed adverse effects on behavior, respiration, or survival [33,34].

Clams and other benthic species represent an important food resource, and seafood industries are vital to Florida’s economy. As such, red tide mitigation products inherently pose potential exposure risks to the food supply and must be assessed for food safety. Aluminum, a nonessential metal, can exert toxic effects when present at elevated concentrations. Furthermore, Al is common in food and pharmaceuticals, with no dietary toxicity having been reported, even under heavy usage of antacids which are known to contain high quantities of Al [43]. MCII-exposed clams in this study contained approximately 12.6–20.0 mg/kg of elemental Al. A review of aluminum concentrations in common foods reported total dietary exposures of 2–160 mg/day, with food additives comprising up to 95 mg/day. In addition, antacid use was shown to cause ingestion of approximately 50–5000 mg/day [43]. Cooking or canning food contributes substantially to dietary aluminum and other studies found commercial bivalves preserved in aluminum cans contained 75 mg/100 g dry weight [44]. Thus, the total Al accumulation in this study does not raise concerns for the health of the clams or the humans who eat them. Future investigations should incorporate a broader range of benthic species to provide greater insight into the effects of MCII treatment and the accumulation of metals, particularly aluminum, in tissue.

## 5. Conclusions

This study served to reinforce the effectiveness of MCII on the removal of *K. brevis* cells via flocculation and aimed to investigate the potential of brevetoxin bioavailability to benthic organisms from settled HAB clay flocs. In this small-scale mesocosm study, a 91% *K. brevis* cell reduction post-treatment was demonstrated, signifying an effective clay flocculation treatment that led to a decrease in waterborne brevetoxins by 50% after 5 h. One-hour post-water exchange, brevetoxin concentrations were minimal in both the control and treated tanks, but subsequent water analyses showed a slight increase in total brevetoxins from the treated tanks with the major contributor being BTX-B5, the oxidized form of BTX-2. From this, MCII-treated sediment samples were shown to contain higher concentrations of brevetoxins at each time point tested when compared to controls, leading to higher concentrations of brevetoxins available in the benthos in a short period of time. Post-treatment analyses of clam tissues revealed that although brevetoxin abundance in the benthos is increased from MCII flocculating *K. brevis* cells, the bioavailability of the brevetoxins to *M. mercenaria* is minimal. Although not statistically significant, at T = 24 a slight increase in measured brevetoxins and their metabolites was observed in the tissues of clams exposed to MCII floc; however, by T = 72, in vivo toxin concentrations were still comparable to controls. Although in vivo brevetoxin concentrations are not significantly increased due to the MCII treatment, aluminum concentrations were on average 5.1-fold higher when compared to controls. Throughout the exposure, no organism deaths occurred. This information, coupled with previous research, led us to the conclusion that the increase in aluminum does not raise concerns about the health of the clams or the humans who eat them. However, further investigation into the effects of aluminum ingestion by *M. mercenaria* and other benthic species is required to support this claim.

An investigation into the pseudofeces produced by *M. mercenaria* and the increased production thereof, presumably from the addition of the MCII treatment, is suggested to determine the true bioavailability of brevetoxins from flocculated *K. brevis* cells. It is presumed that, although toxin concentrations did not notably increase within the test organisms, the increased production of pseudofeces can be attributed to either the higher concentrations of *K. brevis* cells and their brevetoxins within the clay, leading to a more selective feeding regime by the test organisms, or selective rejection of the clay particulate. In addition, the investigation of *K. brevis* cell viability within the clay floc can lead to a better understanding of the half-life of the brevetoxins seen in the water samples. This study serves as a steppingstone for assessing ecosystem effects of HAB mitigation in Florida marine waters utilizing a clay-based treatment and has shed insight onto the fate of brevetoxins and their bioavailability post-clay treatment.

## Data Availability

The original contributions presented in this study are included in the article. Further inquiries can be directed to the corresponding author.

## Supplementary Materials

The following supporting information can be downloaded at: https://www.mdpi.com/article/10.3390/toxins17110560/s1, Figure S1: Mean ± standard deviation of various trace metal concentrations within tissue of treatment (MCII) and control (KBC) groups (*n* = 3 clams per group); Table S1: Target Analyte:Phosophorus (P) ratios expressed in mmol analyte/mol P; Table S2: Target Analyte:Phosophorus (P) ratios expressed in ug analyte/mg P; Table S3: Concentrations of target analyte in dried sample expressed in mg analyte/kg dried sample; Table S4: Reference sample results, certified values and percent recoveries of analytes with reported reference values; Table S5: Reference sample results, certified values and percent recoveries of analytes with reported reference values; Table S6: Sample Names and dry tissue mass (g); Table S7: Multiple reaction monitoring (MRM) parameters used for mass spectrometry analysis of all water and sediment samples; Table S8: LC conditions for water and sediment samples analyzed. The mobile phase consisted of water fortified with 0.1 percent (%) formic acid (FA) (solvent A) and acetonitrile fortified with 0.1% FA (solvent B); Table S9: Multiple reaction monitoring (MRM) parameters used for mass spectrometry analysis of all tissue samples; Table S10: LC conditions for tissue samples analyzed. The mobile phase consisted of water fortified with 0.1 percent (%) formic acid (FA) (solvent A) and acetonitrile fortified with 0.1% FA (solvent B).

## Abbreviations

The following abbreviations are used in this manuscript:

Al	Aluminum
BTX	Brevetoxins
EC_50_	Effective Concentration 50%
GF/F	Glass Microfibre Filter
HAB	Harmful Algal Bloom
ICP-MS	Inductively Coupled Plasma-Mass Spectrometry
LC	Liquid Chromatography
LD_50_	Lethal Dose 50%
MCII	Modified Clay-II
ORP	Oxidation Reduction Potential
PAC	Polyaluminum Chloride
PTFE	Polytetrafluoroethylene
MRM	Multiple Reaction Monitoring
RO	Reverse Osmosis
RTMTDI	Red Tide Mitigation and Technology Development Initiative
SPE	Solid Phase Extraction
TQ	Triple Quadrupole
UHP	Ultra High Purity
UHPLC-MS	Ultra High-Performance Liquid Chromatography-Mass Spectrometry
