# Supplementary material for: Brevetoxin Dynamics and Bioavailability from Floc Following PAC-Modified Clay Treatment of Karenia brevis Blooms
Source: Toxins (Basel). 2025 Nov 13;17(11):560. doi: 10.3390/toxins17110560 (PMC12656167; doi:10.3390/toxins17110560)
Supplement: Supplementary file 1 [file toxins-17-00560-s001.zip › toxins-3936226-supplementary.pdf]

Supporting Information

Brevetoxin Dynamics and Bioavailability from Floc Following PAC-Modified Clay Treatment of *Karenia brevis* Blooms

Nicholas R. Ohnikian[1,†], Christopher D. Sibley[1,†], R. Ben Freiberger[2], Kristen N. Buck[2], Alyssa Myers[1], Samantha Harlow[1], Donald M. Anderson[3], Richard Pierce[1] and Jennifer H. Toyoda[1,*]

1 Ecotoxicology Research Program, Mote Marine Laboratory, Sarasota, FL 34240, USA; nohnikian@mote.org (N.R.O.); csibley@mote.org (C.D.S.); alyssa.meyers@spartans.ut.edu (A.M.); sharlow@mote.org (S.H.); rich@mote.org (R.P.)

2 College of Earth, Ocean, and Atmospheric Sciences, Oregon State University, Corvallis, OR 97331, USA; freiberr@oregonstate.edu (R.B.F.); kristen.buck@oregonstate.edu (K.N.B.)

3 Woods Hole Oceanographic Institution, Woods Hole, MA 02543, USA; danderson@whoi.edu

* Correspondence: jtoyoda@mote.org

† These authors contributed equally to this work.

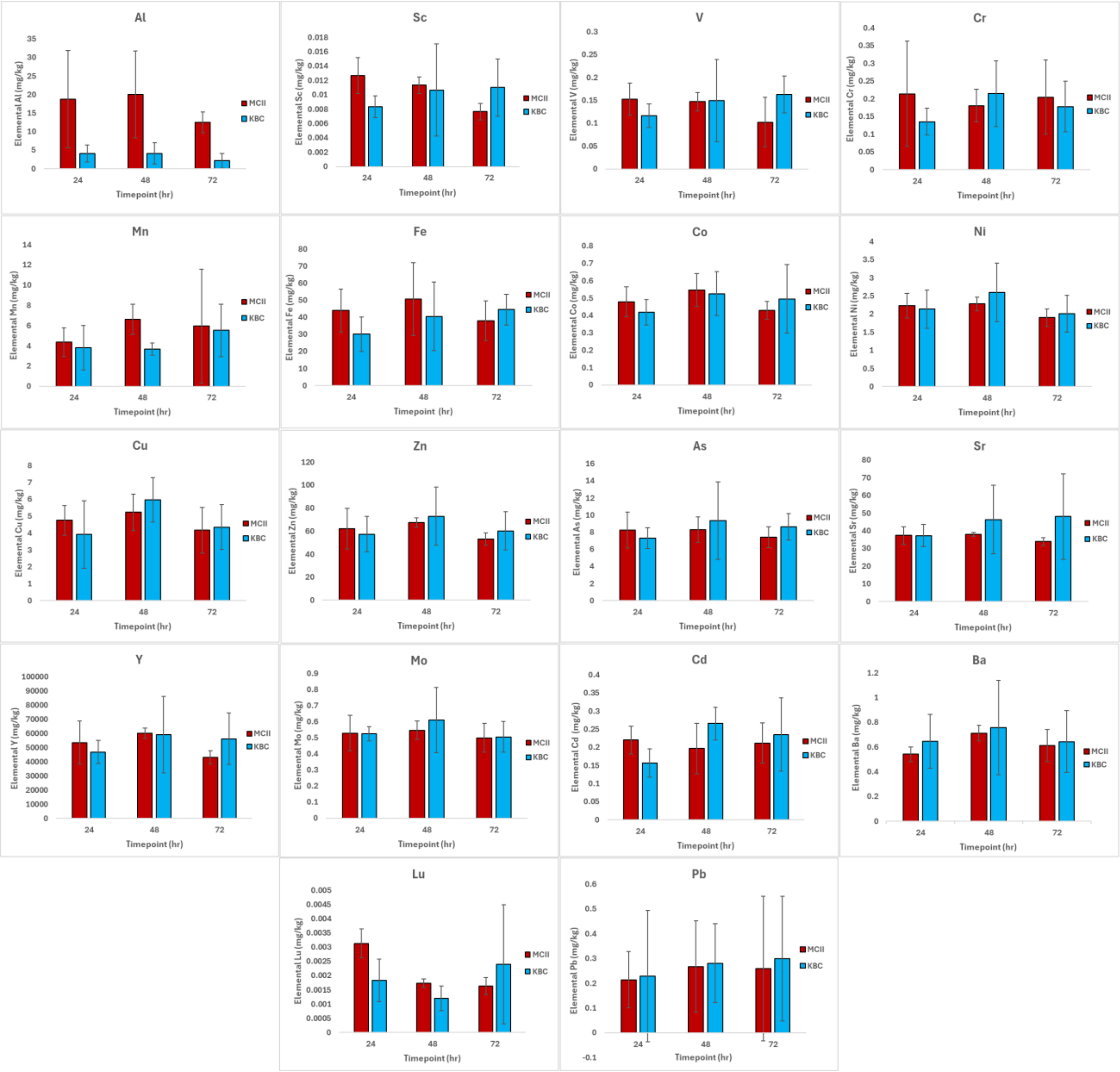

Figure S1. Mean ± standard deviation of various trace metal concentrations within tissue of treatment (MCII) and control (KBC) groups (n = 3 clams per group). Tissue samples were collected at 24-, 48-, and 72-hours post treatment and analyzed by ICP-MS. Concentrations of target analyte are expressed as milligrams of target analyte per kilogram of clam tissue (mg/kg).

**Metal:P ratios (mmol/mol)**	Al	Sc	V	Cr	Mn	Fe	Co	Ni	Cu	Zn	As	Sr	Y	Mo	Cd	Ba	Lu	Pb
KBC 1 4/9/25	0.770	0.062	0.020	0.027	0.690	4.00	0.057	0.315	0.413	7.12	0.877	3.88	4440	0.049	0.013	0.037	0.000	0.003
KBC 2 4/9/25	0.839	0.061	0.016	0.015	0.221	3.60	0.054	0.237	0.346	5.89	0.684	2.95	3840	0.042	0.009	0.029	0.000	0.003
KBC 3 4/9/25	1.47	0.059	0.018	0.020	0.685	4.86	0.056	0.300	0.637	7.43	0.738	3.21	4110	0.039	0.011	0.042	0.000	0.017
KBC 1 4/10/25	0.894	0.060	0.021	0.026	0.274	4.79	0.047	0.246	0.475	6.43	0.811	3.23	4170	0.036	0.010	0.037	0.000	0.004
KBC 2 4/10/25	0.558	0.061	0.015	0.025	0.739	4.39	0.065	0.292	0.675	7.24	0.770	3.15	3770	0.042	0.026	0.038	0.000	0.008
KBC 3 4/10/25	0.799	0.058	0.015	0.026	0.395	4.02	0.061	0.298	0.676	7.34	0.688	3.37	4190	0.042	0.014	0.026	0.000	0.016
KBC 1 4/11/25	0.879	0.052	0.022	0.018	0.781	5.86	0.056	0.255	0.548	6.85	0.877	2.98	4560	0.042	0.010	0.030	0.000	0.005
KBC 2 4/11/25	nda	0.065	0.024	0.024	0.975	5.80	0.046	0.206	0.352	5.34	0.817	5.38	3510	0.040	0.017	0.042	0.000	0.006
KBC 3 4/11/25	1.00	0.056	0.026	0.033	0.482	6.30	0.089	0.319	0.677	8.81	0.911	3.52	6320	0.037	0.019	0.031	0.000	0.021
MCII 1 4/9/25	9.17	0.100	0.023	0.017	0.423	5.83	0.060	0.273	0.563	6.49	0.767	3.37	4200	0.040	0.014	0.033	0.000	0.014
MCII 2 4/9/25	0.788	0.069	0.019	0.036	0.463	5.15	0.048	0.207	0.446	6.17	0.698	2.41	3930	0.033	0.011	0.022	0.000	0.004
MCII 3 4/9/25	5.98	0.073	0.020	0.024	0.763	5.18	0.062	0.327	0.550	6.79	0.802	3.21	4200	0.041	0.015	0.029	0.000	0.005
MCII 1 4/10/25	2.65	0.051	0.024	0.029	0.792	9.46	0.058	0.297	0.619	7.25	0.897	2.99	4840	0.039	0.017	0.040	0.000	0.016
MCII 2 4/10/25	4.52	0.080	0.022	0.020	1.211	4.65	0.068	0.284	0.511	7.87	0.710	3.52	5060	0.042	0.010	0.038	0.000	0.006
MCII 3 4/10/25	7.80	0.082	0.017	0.025	0.661	5.46	0.074	0.262	0.642	7.31	0.788	2.93	4740	0.042	0.011	0.034	0.000	0.006
MCII 1 4/11/25	2.79	0.055	0.022	0.020	1.533	6.34	0.044	0.193	0.595	5.16	0.766	2.85	3150	0.043	0.017	0.036	0.000	0.020
MCII 2 4/11/25	4.41	0.067	0.015	0.022	0.226	4.35	0.066	0.289	0.524	7.26	0.840	2.94	4360	0.036	0.012	0.027	0.000	0.003
MCII 3 4/11/25	3.20	0.076	0.013	0.055	0.628	5.00	0.062	0.285	0.383	6.80	0.695	3.25	3920	0.042	0.014	0.041	0.000	0.004

Table S1. Target Analyte:Phosophorus (P) ratios expressed in mmol analyte/mol P.

Metal:P ratios (ug/mg P)	Al	Sc	V	Cr	Mn	Fe	Co	Ni	Cu	Zn	As	Sr	Y	Mo	Cd	Ba	Lu	Pb
KBC 1 4/9/25	0.671	0.002	0.032	0.046	1.23	7.21	0.108	0.596	0.848	15.0	2.12	11.0	12800	0.153	0.0462	0.166	0.0008	0.021
KBC 2 4/9/25	0.671	0.002	0.026	0.025	0.392	6.49	0.103	0.449	0.709	12.4	1.66	8.35	11000	0.131	0.0316	0.130	0.0004	0.022
KBC 3 4/9/25	0.731	0.002	0.030	0.033	1.22	8.77	0.106	0.568	1.31	15.7	1.78	9.07	11800	0.120	0.0408	0.188	0.0003	0.112
KBC 1 4/10/25	0.779	0.002	0.034	0.043	0.487	8.62	0.089	0.467	0.974	13.6	1.96	9.12	12000	0.113	0.0351	0.162	0.0002	0.028
KBC 2 4/10/25	0.486	0.002	0.025	0.041	1.31	7.91	0.123	0.553	1.39	15.3	1.86	8.90	10800	0.130	0.0952	0.169	0.0003	0.052
KBC 3 4/10/25	0.697	0.002	0.026	0.043	0.701	7.25	0.115	0.565	1.39	15.5	1.66	9.52	12000	0.129	0.0507	0.116	0.0002	0.104
KBC 1 4/11/25	0.767	0.002	0.036	0.030	1.39	10.6	0.106	0.484	1.13	14.5	2.12	8.44	13100	0.130	0.0368	0.131	0.0015	0.034
KBC 2 4/11/25	nda	0.003	0.039	0.041	1.73	10.5	0.087	0.391	0.722	11.3	1.98	15.2	10100	0.124	0.0603	0.187	0.0002	0.041
KBC 3 4/11/25	0.874	0.002	0.042	0.056	0.854	11.4	0.170	0.605	1.39	18.6	2.20	9.95	18200	0.115	0.0689	0.139	0.0003	0.138
MCII 1 4/9/25	7.99	0.004	0.038	0.029	0.750	10.5	0.114	0.517	1.16	13.7	1.86	9.54	12100	0.125	0.0513	0.148	0.0010	0.095
MCII 2 4/9/25	0.687	0.002	0.031	0.061	0.821	9.29	0.091	0.392	0.92	13.0	1.69	6.81	11300	0.104	0.0412	0.096	0.0005	0.029
MCII 3 4/9/25	5.21	0.003	0.033	0.040	1.35	9.34	0.119	0.619	1.13	14.3	1.94	9.08	12100	0.126	0.0561	0.127	0.0007	0.031
MCII 1 4/10/25	2.31	0.002	0.039	0.050	1.41	17.1	0.111	0.563	1.27	15.3	2.17	8.44	13900	0.120	0.0629	0.179	0.0004	0.110
MCII 2 4/10/25	3.94	0.003	0.036	0.034	2.15	8.39	0.130	0.538	1.05	16.6	1.72	9.97	14500	0.131	0.0358	0.169	0.0004	0.038
MCII 3 4/10/25	6.79	0.002	0.029	0.043	1.17	9.85	0.141	0.497	1.32	15.4	1.91	8.29	13600	0.131	0.0383	0.152	0.0004	0.038
MCII 1 4/11/25	2.43	0.002	0.036	0.033	2.72	11.4	0.085	0.366	1.22	10.9	1.85	8.08	9030	0.134	0.0608	0.159	0.0003	0.132
MCII 2 4/11/25	3.84	0.002	0.024	0.036	0.402	7.84	0.126	0.547	1.08	15.3	2.03	8.32	12500	0.112	0.0450	0.121	0.0005	0.022
MCII 3 4/11/25	2.79	0.002	0.019	0.101	1.12	9.03	0.117	0.538	0.78	14.3	1.68	9.17	11200	0.130	0.0515	0.183	0.0006	0.027

Table S2. Target Analyte:Phosophorus (P) ratios expressed in ug analyte/mg P.

**mg analyte/kg sample**	Al	P	Sc	V	Cr	Mn	Fe	Co	Ni	Cu	Zn	As	Sr	Y	Mo	Cd	Ba	Lu	Pb
KBC 1 4/9/25	2.48	3420	0.008	0.111	0.157	4.19	24.7	0.370	2.04	2.90	51.4	7.26	37.6	43700	0.525	0.158	0.567	0.0027	0.071
KBC 2 4/9/25	2.97	3700	0.007	0.094	0.091	1.45	24.0	0.381	1.66	2.62	46.0	6.12	30.8	40700	0.483	0.117	0.480	0.0014	0.080
KBC 3 4/9/25	6.70	4770	0.010	0.145	0.157	5.80	41.8	0.504	2.71	6.23	74.8	8.51	43.3	56200	0.570	0.195	0.894	0.0014	0.535
KBC 1 4/10/25	7.32	7380	0.018	0.252	0.317	3.59	63.7	0.657	3.44	7.19	100	14.5	67.4	88500	0.831	0.259	1.199	0.0017	0.208
KBC 2 4/10/25	1.73	3300	0.006	0.083	0.136	4.32	26.1	0.406	1.83	4.57	50.4	6.14	29.4	35700	0.429	0.314	0.558	0.0009	0.172
KBC 3 4/10/25	3.41	4440	0.008	0.113	0.190	3.11	32.2	0.512	2.51	6.16	68.7	7.38	42.3	53300	0.573	0.225	0.515	0.0010	0.463
KBC 1 4/11/25	2.62	3240	0.007	0.117	0.098	4.49	34.2	0.344	1.57	3.64	46.8	6.87	27.3	42400	0.420	0.119	0.424	0.0048	0.110
KBC 2 4/11/25	nda	4900	0.015	0.191	0.200	8.47	51.3	0.425	1.91	3.54	55.3	9.69	74.5	49400	0.609	0.295	0.917	0.0010	0.201
KBC 3 4/11/25	3.83	4240	0.011	0.180	0.236	3.62	48.2	0.718	2.56	5.88	78.8	9.34	42.2	76900	0.489	0.292	0.590	0.0014	0.585
MCII 1 4/9/25	30.7	3590	0.015	0.137	0.105	2.70	37.7	0.408	1.85	4.15	49.2	6.66	34.2	43300	0.447	0.184	0.530	0.0036	0.340
MCII 2 4/9/25	4.67	6310	0.013	0.193	0.383	5.18	58.6	0.573	2.47	5.77	82.2	10.6	43.0	71100	0.654	0.260	0.605	0.0032	0.186
MCII 3 4/9/25	21.0	3840	0.010	0.128	0.154	5.20	35.9	0.456	2.38	4.33	55.1	7.45	34.9	46300	0.485	0.216	0.489	0.0026	0.117
MCII 1 4/10/25	10.8	4350	0.010	0.170	0.215	6.12	74.3	0.481	2.45	5.53	66.6	9.44	36.7	60400	0.524	0.274	0.780	0.0019	0.479
MCII 2 4/10/25	16.0	3860	0.012	0.138	0.129	8.29	32.4	0.501	2.08	4.04	64.1	6.62	38.5	56100	0.506	0.138	0.651	0.0016	0.147
MCII 3 4/10/25	33.3	4660	0.012	0.133	0.198	5.46	45.9	0.656	2.32	6.13	71.9	8.88	38.6	63400	0.612	0.178	0.708	0.0017	0.175
MCII 1 4/11/25	11.2	4520	0.009	0.160	0.148	12.3	51.6	0.382	1.65	5.52	49.2	8.37	36.5	40800	0.603	0.275	0.715	0.0013	0.597
MCII 2 4/11/25	15.8	3870	0.007	0.095	0.140	1.56	30.4	0.486	2.12	4.16	59.3	7.87	32.2	48500	0.432	0.174	0.467	0.0019	0.084
MCII 3 4/11/25	10.7	3590	0.007	0.052	0.326	4.00	32.3	0.421	1.94	2.82	51.4	6.03	33.0	40300	0.466	0.187	0.654	0.0017	0.097

Table S3. Concentrations of target analyte in dried sample expressed in mg analyte/kg dried sample.

	Sc (mg/kg)	V (mg/kg)	Cr (mg/kg)	Mn (mg/kg)	Fe (mg/kg)	Co (mg/kg)	Ni (mg/kg)	Cu (mg/kg)	Zn (mg/kg)	As (mg/kg)	Sr (mg/kg)	Mo (mg/kg)	Cd (mg/kg)	Pb (mg/kg)
BCR-414 Average (n=4)	0.31	5.85	17.5	248.	1547	1.33	16.0	27.5	104	6.97	244	1.32	0.345	3.71
Standard Deviation	0.05	0.23	1.65	2.45	25.7	0.01	0.99	0.19	1.20	0.17	2.59	0.15	0.004	0.30
Certified Value (mg/kg)	0.54	8.10	23.8	299	1850	1.43	18.8	29.5	112	6.82	261	1.35	0.38	3.97
Percent Recovery	58.2	72.3	73.7	83.0	83.6	93.3	84.9	93.2	93.3	102.1	93.5	97.6	90.2	93.3

Table S4. Reference sample results, certified values and percent recoveries of analytes with reported reference values.

	Al (mg/kg)	P (mg/kg)	V (mg/kg)	Cr (mg/kg)	Mn (mg/kg)	Fe (mg/kg)	Co (mg/kg)	Ni (mg/kg)	Cu (mg/kg)	Zn (mg/kg)	As (mg/kg)	Sr (mg/kg)	Mo (mg/kg)	Cd (mg/kg)	Ba (mg/kg)	Pb (mg/kg)
DORM-5 Average (n=4)	125	4856	0.13	0.36	0.60	69.6	0.05	0.34	2.21	23.4	10.4	7.76	0.11	0.12	0.20	0.05
Standard Deviation	12.7	608	0.01	0.09	0.09	9.09	0.01	0.04	0.28	2.56	1.37	1.21	0.01	0.02	0.03	0.01
Certified Value (mg/kg)	250	6230	0.35	0.52	1.06	1113	0.06	0.44	3.30	28.7	13.3	9.87	0.13	0.14	0.40	0.06
Percent Recovery	50.1	77.9	38.4	69.2	56.8	61.6	78.2	76.3	67.1	81.7	78.1	78.7	81.1	77.9	49.8	90.8

Table S5. Reference sample results, certified values and percent recoveries of analytes with reported reference values.

Sample Name	Dry Mass (g)
KBC 1 4/9/25	0.0960
KBC 2 4/9/25	0.1041
KBC 3 4/9/25	0.0992
KBC 1 4/10/25	0.0834
KBC 2 4/10/25	0.1089
KBC 3 4/10/25	0.0779
KBC 1 4/11/25	0.1331
KBC 2 4/11/25	0.0775
KBC 3 4/11/25	0.0925
MCII 1 4/9/25	0.0916
MCII 2 4/9/25	0.0820
MCII 3 4/9/25	0.0790
MCII 1 4/10/25	0.0990
MCII 2 4/10/25	0.0842
MCII 3 4/10/25	0.0917
MCII 1 4/11/25	0.0863
MCII 2 4/11/25	0.1018
MCII 3 4/11/25	0.0767

Table S6. Sample Names and dry tissue mass (g).

Compound	Start time (min)	End Time (min)	Precursor (m/z)	Product (m/z)	Collision Energy (V)	Min Dwell Time (ms)
BTX-1	8.1	10	867.4	403.179	20	59.94
BTX-1	8.1	10	867.4	831.429	18.98	59.94
BTX-1	8.1	10	867.4	849.458	13.3	59.94
BTX-2	8.1	10	895.4	751.417	17.59	59.94
BTX-2	8.1	10	895.4	859.458	20.08	59.94
BTX-2	8.1	10	895.4	877.458	14.69	59.94
BTX-3	6	8	897.4	725.375	19.83	65.177
BTX-3	6	8	897.4	807.458	11.66	65.177
BTX-3	6	8	897.4	825.458	10.73	65.177
BTX-B5	6	8	911.4	857.417	19.7	65.177
BTX-B5	6	8	911.4	875.458	18.98	65.177
BTX-B5	6	8	911.4	893.458	17.17	65.177

Table S7. Multiple reaction monitoring (MRM) parameters used for mass spectrometry analysis of all water and sediment samples.

No	Time (min)	Flow (mL/min)	%B
1	0.000	0.200	50.0
2	1.000	0.200	50.0
3	10.000	0.200	95.0
4	11.000	0.200	50.0
5	15.000	0.200	50.0

Table S8. LC conditions for water and sediment samples analyzed. The mobile phase consisted of water fortified with 0.1 percent (%) formic acid (FA) (solvent A) and acetonitrile fortified with 0.1% FA (solvent B).

Compound	Retention time (min)	RT Window (min)	Precursor (m/z)	Product (m/z)	Collision Energy (V)	Min Dwell Time (ms)
Cystine-BTX-2 Sulfoxide	6.5	3	1034.52	753.6	35	131.269
Cystine-BTX-2 Sulfoxide	6.5	3	1034.52	929.4	35	131.269
Cystine-BTX-2	6.9	3	1018.6	204.2	50	131.269
Cystine-BTX-2	6.9	3	1018.6	248.2	50	131.269
Cystine-BTX-1 Sulfoxide	7.36	3	1006.54	901	30	131.269
Cystine-BTX-1	7.68	2.5	990.51	901	30	131.269
BTX-B5	9.42	0.8	911.5	875.5	21	198.269
BTX-3	9.55	0.8	897.4	725.375	19.83	198.269
BTX-3	9.55	0.8	897.4	807.458	11.66	198.269
BTX-3	9.55	0.8	897.4	825.458	10.73	198.269

Table S9. Multiple reaction monitoring (MRM) parameters used for mass spectrometry analysis of all tissue samples.

No	Time (min)	Flow (mL/min)	%B
1	0.000	0.600	30.0
2	1.000	0.600	30.0
3	7.000	0.600	60.0
4	10.000	1.200	95.0
5	20.100	1.200	95.0
6	25.000	0.600	30.0

Table S10. LC conditions for tissue samples analyzed. The mobile phase consisted of water fortified with 0.1 percent (%) formic acid (FA) (solvent A) and acetonitrile fortified with 0.1% FA (solvent B).
